# Misinformation and Public Health Messaging in the Early Stages of the Mpox Outbreak: Mapping the Twitter Narrative With Deep Learning

**DOI:** 10.2196/43841

**Published:** 2023-06-06

**Authors:** Andy Edinger, Danny Valdez, Eric Walsh-Buhi, Jennifer S Trueblood, Lorenzo Lorenzo-Luaces, Lauren A Rutter, Johan Bollen

**Affiliations:** 1 Center for Social and Biomedical Complexity Indiana University Bloomington, IN United States; 2 Cognitive Science Program Indiana University Bloomington, IN United States; 3 Department of Applied Health Science School of Public Health Indiana University Bloomington, IN United States; 4 Department of Psychological and Brain Sciences Indiana University Bloomington, IN United States

**Keywords:** COVID-19, deep learning, misinformation, monkeypox, mpox, outbreak, public health, social media, Twitter

## Abstract

**Background:**

Shortly after the worst of the COVID-19 pandemic, an outbreak of mpox introduced another critical public health emergency. Like the COVID-19 pandemic, the mpox outbreak was characterized by a rising prevalence of public health misinformation on social media, through which many US adults receive and engage with news. Digital misinformation continues to challenge the efforts of public health officials in providing accurate and timely information to the public. We examine the evolving topic distributions of social media narratives during the mpox outbreak to map the tension between rapidly diffusing misinformation and public health communication.

**Objective:**

This study aims to observe topical themes occurring in a large-scale collection of tweets about mpox using deep learning.

**Methods:**

We leveraged a data set comprised of all mpox-related tweets that were posted between May 7, 2022, and July 23, 2022. We then applied Sentence Bidirectional Encoder Representations From Transformers (S-BERT) to the content of each tweet to generate a representation of its content in high-dimensional vector space, where semantically similar tweets will be located closely together. We projected the set of tweet embeddings to a 2D map by applying principal component analysis and Uniform Manifold Approximation Projection (UMAP). Finally, we group these data points into 7 topical clusters using k-means clustering and analyze each cluster to determine its dominant topics. We analyze the prevalence of each cluster over time to evaluate longitudinal thematic changes.

**Results:**

Our deep-learning pipeline revealed 7 distinct clusters of content: (1) cynicism, (2) exasperation, (3) COVID-19, (4) men who have sex with men, (5) case reports, (6) vaccination, and (7) World Health Organization (WHO). Clusters that largely communicated erroneous or irrelevant information began earlier and grew faster, reaching a wider audience than later communications by official instances and health officials.

**Conclusions:**

Within a few weeks of the first reported mpox cases, an avalanche of mostly false, misleading, irrelevant, or damaging information started to circulate on social media. Official institutions, including the WHO, acted promptly, providing case reports and accurate information within weeks, but were overshadowed by rapidly spreading social media chatter. Our results point to the need for real-time monitoring of social media content to optimize responses to public health emergencies.

## Introduction

### Overview

Shortly after adopting a “learn to live with” approach to COVID-19, the World Health Organization (WHO) declared mpox, a viral zoonotic disease caused by the mpox virus, an international health concern [1]. Though previously a relatively rare disease isolated to portions of western and southern Africa, a cluster of cases linked to communities of men who have sex with men (MSM) in parts of Europe and the United States implicated disease spread to communities previously unaffected by the virus [2]. After cases spread to children and adults, regardless of demographics [3], it became clear that interhuman transmission was responsible for most new cases, prompting renewed concerns of a follow-up uncontrollable disease [4].

Similar to the COVID-19 pandemic, news and updates regarding the mpox outbreak spread through mainstream media and social media platforms. However, social media is vulnerable to misinformation that can influence public attitudes toward mpox [5,6]. Given the volume of social media data pertaining to mpox, novel approaches in computational informatics and data science may provide an effective way to monitor public discourse on social media at large scale. In this study, we used a deep-learning approach to examine the evolution of mpox-related narratives on Twitter between May 7, 2022, and July 23, 2022. We present key insights into the public’s consumption of news in the United States, similarities between public reactions to mpox and COVID-19, and the potential application of computational approaches toward verifying findings from previous quantitative and qualitative studies.

### News Consumption, Social Media, and Misinformation

In 2022, public reports and social commentaries regarding global mpox cases became a prevalent component of ongoing news cycles. These reports and news broadcasts echo those presented during the initial stages of the COVID-19 pandemic, which emphasized disease spread, preparation, infection risk, and mitigation strategies [7]. However, research conducted since the COVID-19 pandemic and its early reports reveal that public health outlets have implemented “lessons learned” related to (1) the importance of efficiently disseminating timely and accurate information and (2) the difficulty faced by federal agencies and news outlets in countering misinformation and its impact on people’s perceptions of COVID-19 [8]. This urgency is partially due to the widely documented information overload and incongruent outlets where individuals receive news and health-related information, including social media and social networking websites such as Twitter, Reddit, Instagram, TikTok, and others.

It is not surprising that social media has become a primary outlet for news distribution and internetwork commentating [9], as it has evolved beyond its social connection roots over the last decade. Current estimates suggest that 70% of the US adult population regularly uses at least one social media platform daily, and a significant proportion report being on the internet constantly. Moreover, marginally less than half of US adults (48%) report “often” or “sometimes” getting their news from social media [10], with increasing proportions of the US population entirely disengaged from print or broadcast news [11]. However, it is widely known that information on social media carries a higher risk of misinformation compared to print and broadcast media [5,6]. This risk is particularly high when content is perceived as having a political undertone or motivation [2,12,13] or when digital media literacy is low. For instance, Guess and colleagues [14] asserted that many social media users with low digital literacy skills are ill-equipped to distinguish between low-quality and high-quality news in a global media literacy intervention [14]. A 2017 Pew Research study found that 17% of working-age adults lack digital literacy skills, and digital literacy rates vary greatly depending on key demographics [15].

The rise of curated feeds tailored for each individual social media user has made it all the more challenging to discern or counter misinformation on social media [16]. Social media algorithms now customize feeds to fit the digital footprint of the user, resulting in a higher probability that a person will receive content from individuals they already agree with or find entertaining. These individualized feeds, known as echo chambers or filter bubbles [17], may encourage people to spend more time on social media, reinforcing its addictive properties. However, these feeds also create the impression that a single user-approved perspective is accurate or correct by not presenting other angles or perspectives on an issue. Echo chambers have been widely credited with fueling the political divide in the United States and abroad [18]. For example, during COVID-19, political misinformation often framed the disease as fabricated, a profiteering effort by the federal government, and a scapegoat to inoculate people with a purportedly fake vaccine [19]. There is evidence to suggest that similar political undertones may also be promoting misinformed perspectives about mpox among certain echo chambers or people with specific political predispositions. Nonetheless, more research is needed to investigate this topic.

### Mass Communication Similarities and Differences Between COVID-19 and Mpox

Although social reactions to the mpox outbreak and the COVID-19 pandemic share many similarities, mpox has unique characteristics that distinguish it from COVID-19. COVID-19 infection yields flu-like symptoms, such as headache, cough, fatigue, body aches, and general congestion in mild cases, while in severe cases, it can lead to acute respiratory distress syndrome and other potentially fatal co-occurring outcomes [20]. On the other hand, although mpox is rarely fatal, it produces flu-like symptoms in addition to observable boils and other lesions that accompany the infection [2], a feature that is unique to pox-family viruses. In contrast to COVID-19, early mpox cases were attributed to MSM communities [21], and a Food and Drug Administration (FDA)–approved vaccine already existed for mpox during the pandemic’s onset, whereas no viable vaccine existed for COVID-19 [22].

The unique characteristics of mpox as compared to COVID-19 could potentially foster different public reactions to each disease. For instance, the differences could potentially frame mpox as less threatening or severe than the COVID-19 pandemic [23]. The differences could also suggest that mpox only affects certain populations, highlighting the stigma and prejudice that may accompany mpox infection [24]. Indeed, a prevailing misconception surrounding mpox is that it is a sexually transmitted infection exclusively affecting MSM communities [25]. However, as a touch-borne disease, mpox is easily transmissible through any form of social contact, regardless of gender or sexual orientation. Evidence suggests this stigma is already having negative health outcomes among queer and race-minority communities. For instance, Owens and Huback [26] contend that sexual and gender minorities and people assigned male at birth perceive societal stigmatization based on the larger mpox narrative [26]. Furthermore, the outdated and former name of the infection (ie, monkeypox) perpetuated racist tropes, as pointed out by Damaso [27].

Research on social media’s role in shaping perceptions and behaviors during the mpox outbreak highlights the presence of prejudices and stereotypes. One study found that stigmatizing beliefs about mpox can hinder individuals from following recommended guidelines, such as vaccination uptake, handwashing, and social distancing [28]. While similar misperceptions about COVID-19 also impeded adherence to social distancing measures, the stigma associated with mpox may exacerbate the issue by perpetuating harmful stereotypes or promoting conspiracy theories. For example, Zenone and Caulfield [29] identified 11 categories of conspiracy theories related to the mpox outbreak in short-form social media videos. In addition, Anoop and Sreelakshmi [30] analyzed Reddit comments and found that while some posts provided helpful information on symptoms, transmission risk, and travel warnings, others exhibited stigmatizing biases that stem from a fear of the unknown.

### Addressing the Need for Longitudinal, Computationally Driven Analyses of Mpox

Extensive research has been conducted on mpox-related social stigma, knowledge, and attitudes, as well as qualitative assessments of mpox content on social media. However, these studies may have limitations, such as cross-sectional designs or small sample sizes. Studies suggest that interventions, whether conducted in person or on the internet, can reduce biases associated with mpox among study participants. Nonetheless, the prevalence of misinformation and fake news on social media necessitates further analyses that can offer a more nuanced understanding of mpox dialogues on these platforms. Valdez and Patterson [31] propose that computationally driven analyses can complement and verify traditional quantitative or qualitative research findings. Therefore, this study aims to investigate longitudinal mpox-related narratives on social media using deep-learning techniques.

### This Study

The mpox outbreak marks the first public health emergency and response following a once-in-a-generation global pandemic. While the dissemination of medically accurate facts and information about mpox may have helped quell public anxieties about infection rates and spread, the outbreak is not immune to misinformation in the digital space. Therefore, this study aimed to generate themes from a collection of tweets specifically pertaining to mpox and examine how the narrative of the outbreak evolved over time. Our study was guided by 3 research questions (RQs):

- RQ1: What themes emerge from a deep-learning analysis of mpox-related tweets?
- RQ2: How do themes identified from a deep-learning model evolve over the course of the mpox outbreak?
- RQ3: What do these themes collectively imply about public health responses during global public health emergencies?

The findings from our study have the potential to inform the extent to which lessons learned during the COVID-19 pandemic can be applied to future public health emergencies. Examining the relative difference between medically accurate information and misinformation’s noise may also highlight the ongoing risk that social media poses in shaping norms, attitudes, and behaviors toward disease outbreaks and associated responses.

## Methods

### Data

Our analysis relied on a publicly available repository of 254,363 tweet IDs (“Mpox2022Tweets”) related to the 2022 mpox outbreak [32]. We retrieved the full tweet content for each ID provided on August 15, 2022, using the Twitter application programming interface, yielding 230,163 tweets posted between May 7, 2022, and July 23, 2022 (meaning 24,200 were deleted or otherwise unavailable). Given the need to rapidly produce deep-learning models for a collection of tweets, we deemed a manual evaluation of tweets with robust forms of qualitative inquiry impractical. We thus designed our analysis to reveal the structure of the entirety of web-based discussions surrounding the mpox outbreak with a computational pipeline designed to parse tweets into core themes.

### Analysis

#### Overview

To analyze our data, we applied the following analytic pipeline: (1) calculate vectors using the Sentence Bidirectional Encoder Representations From Transformer (S-BERT) algorithm and (2) data visualization using a principal component analysis (PCA) with Uniform Manifold Approximation (UMAP).

#### Framework of S-BERT

S-BERT is an extension of the state-of-the-art Bidirectional Encoder Representations From Transformers (BERT) algorithm, which applies neural networks to detect patterns in large-scale text data [33,34]. The BERT family of algorithms is trained on large-scale text corpora and can generate numerical vectors for texts that allow the evaluation of their semantic similarity to other texts [35]. S-BERT is specifically designed for the comparison of semantic information on the sentence level rather than the word or token level. Given the goal of analyzing topical differences across tweets, this focus on longer language samples allows for better comparison of the similarities and differences between various input tweets.

#### PCA and UMAP Techniques

Text vectors calculated using the S-BERT algorithm are highly dimensional and complex. For visualization purposes, we used a combination of PCA and UMAP to reduce their dimensionality to 2 dimensions [36-39]. PCA and UMAP are common techniques applied for these purposes. PCA extracts the principal components, variables which maximally capture the variance of the data set. By projecting data on its principal components, it is possible to optimally represent the most significant variance in the data set in minimal dimensions. UMAP reduces data dimensionality while preserving the distance between each data point and its neighbors. By varying the parameters of the UMAP algorithm, one may control the emphasis on preservation of local versus global structure. Through experimentation with these parameter values, it is possible to optimize the UMAP reduction process to preserve the similarity measures that are represented in S-BERT’s high-dimensional vector spaces.

#### K-Means Clustering

K-means clustering is an algorithm designed to partition data into a predefined number (K) of optimally dense subsets of data points [40]. K-means initially assigns a set of K random cluster centers, assigning each datapoint to the cluster whose center is closest in space. The algorithm then iteratively adjusts the center points, minimizing the distance between the center and the assigned data, until a set of optimally dense clusters is found. The addition of the k-means clustering algorithm to our analysis pipeline facilitates identification of topical clusters for further investigation.

### Procedure

We leveraged S-BERT, PCA, and UMAP to place each tweet in a visual map highlighting the structure of topic distributions within the total volume of mpox-related messages in our data. To allow visualization, we first reduced the data set to a randomly selected sample of 10% of all tweets retrieved, producing a final set of 17,646 tweets for analysis. This random sampling reduces the number of tweets to visualize while maintaining the original topic, content, and origin distributions. We then mapped each tweet in our sample into a 384-dimensional vector produced by S-BERT. These vectors provide numerical representations of each tweet’s content, so we can calculate the similarity of any pair of tweets from the degree to which their S-BERT vectors align.

To visualize our data in a 2D map, we reduced the dimensionality of the S-BERT vectors to 2 dimensions with (1) PCA to retain only the 40 most important components of the initial analysis and (2) UMAP to project each of the tweet vectors onto a 2D plane (our map). We then grouped tweets into topical clusters using a k-means clustering algorithm based on their position in the map. Silhouette testing indicated that 7 k-means clusters were optimal for separation of distinct topics, which we confirmed through our own examination of the data. We constructed an interactive map of the tweets, allowing the contents of tweets within each topic cluster to be viewed.

We independently assessed the clusters with an informal qualitative thematic assessment, providing topic labels and contextualization of their meaning within the larger mpox narrative. A review and discussion of each cluster yielded consensus cluster labels, as shown in Table 1. Indeed, each member of the study team reviewed a subset of posts from each cluster and identified recurring themes or ideas. The research team was also interested if clusters (1) exhibited indications of misinformation and (2) contained evidence of jokes, insincere comments, or general snarkiness inherent to social media data. These 2 considerations influenced the naming of each cluster, such that jokes and sarcasm influenced the naming outcome. For example, if 1 cluster had many tweets containing a shared news story, yet the remaining part of the tweet contained evidence of a joke, sarcasm, anger, or frustration, then we would conclude the tweet may fall under a hypothetical “joke” or “frustration” category. However, if a body of tweets contained a shared new story with either (1) no further comment or (2) helpful suggestions (ie, get your vaccination here), then we would conclude the cluster was either directly relevant to health promotion or pertained exclusively to news sharing. Though the naming process follows the nature of the method employed here as well as the various statements contained within each tweet, cluster topics are not exclusive but rather reflect the general thematic trend within each cluster. This is a natural limitation to computational analyses, namely that human language and user-generated social media content are much more complex than the effectiveness of these analyses at categorizing text into mutually exclusive categories. As such, some of our topics contained marginal degrees of overlap (eg, a sarcastic comment responding to news shared by the WHO); however, this is largely consistent with other similar analyses (see Russell et al [41]).

**Table 1:** Summary of cluster content, delineated by cluster name, total retweets (RT), average retweets per cluster (Rc), and example tweets. Due to Twitter’s terms of service restrictions, tweets outlined in our table have been abridged to prevent identification.

Cluster name	RT	Rc	Tweets, n	Sample tweets
Cynicism	91,483	29.68	3082	“Just wait, 2 years from now we’ll have Omega Mpox -- Mpox isn’t airborne, so far as I know, there’s that ‘emergency’ -- So many other illnesses happen every day yet those don’t get sensationalized”
Exasperation	17,495	3.76	4649	“Twitter is loving the Mpox doom narrative, huh? -- Biden liked it so much now he’s leading with a Mpox opening act! -- Here we go again with another breaking emergency like we aren’t all tired”
COVID-19	20,939	7.37	2840	“Mpox time! Get that toilet paper back on your face! -- Never forget that the government and media played into COVID-19 for hysteria -- Joe Biden mentioned we need more money for another pandemic”
MSM^a^	132,987	60.61	2194	“So this is like the HIV epidemic of the 80s huh? -- I guess I have nothing to worry about, this is a gay people thing -- So are you saying Mpox is a homosexual thing?”
Case reports	65,741	36.24	1814	“First Mpox case identified in NY, patient is stable -- Mpox detected in at least seven states, spread likely -- Mpox spreads to US: How to stay informed.”
Vaccination	22,733	13.06	1740	“The good news is a safe and effective vaccine exists, get yours today. -- If you are at high risk for developing Mpox, get your vaccine here -- Vaccines are the best way to protect you and yours from Mpox spread”
WHO^b^	97,591	73.54	1327	“WHO now says Mpox is an international health emergency -- Need Mpox resources? Read the WHO official statement -- WHO declares highest alert for an outbreak”

^a^MSM: men who have sex with men.

^b^WHO: World Health Organization.

### Ethical Considerations

This study represents a secondary data analysis of publicly available social media data. This study and the majority of social media studies following a similar methodological pipeline were exempt from review by the institutional review board. Regardless, all data were scrubbed of any personally identifiable information prior to data cleaning, analysis, and interpretation, and stored on secure, access-limited, encrypted systems to safeguard privacy.

## Results

### Overview

Our study applied deep-learning models to identify themes embedded within a collection of mpox-related tweets. Broadly, we observed a wide array of topics pertaining to both accurate health messaging and misinformed perspectives. We outline key results below without comment.

### RQ1: What Themes Emerge From a Deep-Learning Analysis of Mpox-Related Tweets?

Our deep-learning model revealed 7 clusters embedded within our data. Table 1 provides a summary of cluster composition, including the total number of tweets per cluster, average retweets, and example tweets deemed most illustrative of each cluster’s content. Within the first 4 clusters, we observed high levels of inaccurate information and cynicism regarding news of “yet another international health concern.” Conversely, the latter clusters, including case reports, vaccination, and WHO, seemed to contain accurate health messaging about mpox, including information about transmission and protection, less joke sharing, and more indications for people taking these reports seriously. Although tweets about vaccination campaigns and case reports (and any content generally originating from public health outlets such as the WHO and Centers for Disease Control and Prevention (CDC) were retweeted extensively, these rates lagged other potentially misleading information in topic clusters, such as MSM transmission misinformation and cynicism with repeated health crises.

### RQ2: How Do Themes Identified From a Deep-Learning Model Evolve Over the Course of the Mpox Outbreak?

**Figure 1:**
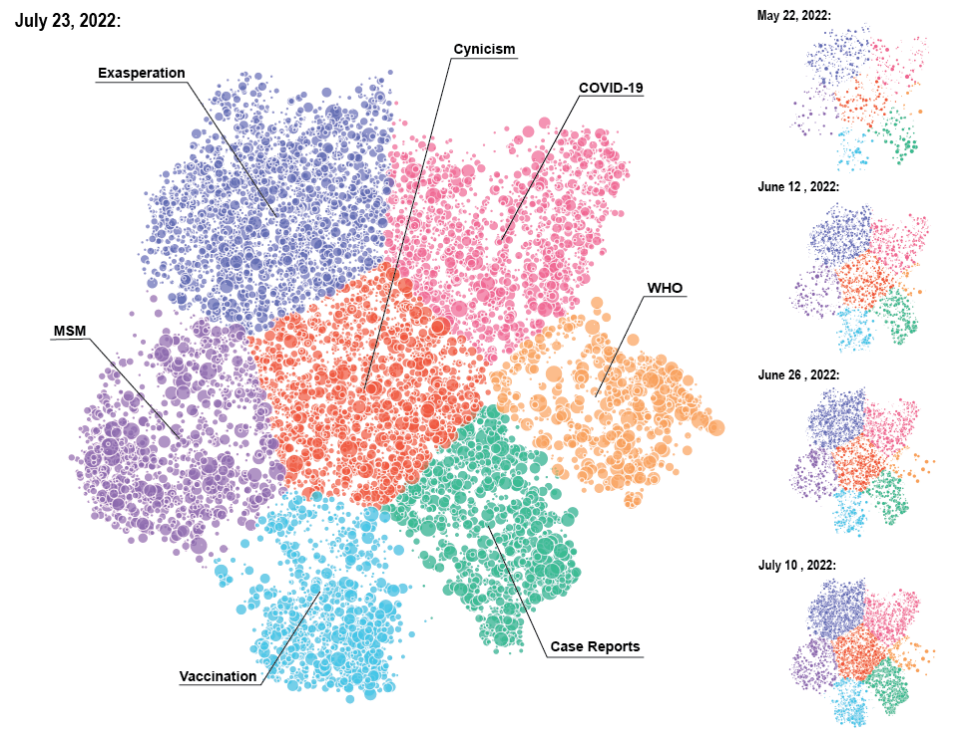
Topic map shows how tweets are grouped spatially according to their content similarities in k=7 clusters. In clockwise order, we find clusters related to “Exasperation” (emergency fatigue, blue), “COVID-19” (comparison with, pink), “WHO” (official declarations by the WHO, yellow), “Case Reports” (green), “Vaccination” (campaigns and availability, light blue), and “MSM transmission” (among men who have sex with men, purple). In the center of these clusters, we find a large cluster of tweets expressing “cynicism” (about mpox messaging, orange). Panels on the right display the cumulative volume of tweets for the specific content clusters over the time period beginning on May 7 and ending on the date shown. The diameter of each point in the map is scaled as a function of the number of times the tweet was “retweeted” and “liked,” such that larger circles indicate more frequent retweets and likes. MSM: men who have sex with men; WHO: World Health Organization.

Figure 1 shows a visual map where each tweet is represented by a small circle positioned in the vicinity of other tweets that are similar in content; coordinates were obtained from a 2D projection of highly dimensional tweet content vectors (see Methods section). Despite the variety of content expected from tens of thousands of tweets posted over a period of 2 months, 7 distinct clusters emerged over time, demonstrating a gradual evolution in mpox social media discourse. Indeed, observed activity patterns in each topical cluster changed considerably over time as the mpox outbreak and associated narratives evolved. We therefore show the evolution of topical activity over time in the right panel of Figure 1 by highlighting evolution and growth of clusters per 2-week period, which reflects changes and evolution of themes over time.

We observed that clusters containing public reactions and misinformation about mpox transmissibility in the MSM community emerged first and dominated activity throughout the period of analysis. These early clusters again implicated general public exhaustion and skepticism of what were perceived to be repeated and fabricated global health emergencies post COVID-19 (tweet: “Here we go again with another scamdemic!”). We also observed higher rates of sarcasm and humor in these clusters, which illustrate the public’s limited ability to internalize the potential threat mpox posed across populations (tweet: “DUDE. Now we have to worry about monkeys, too?”). Only after weeks of unmitigated information spread did topics specific to WHO guidelines, recommendations, and otherwise responsible health messaging emerge and become more prominent. In other words, social media noise and irrelevant content preceded official social media–driven public health responses.

### RQ3: What Do These Themes Imply Collectively About Public Health Responses Amid Global Public Health Emergencies?

**Figure 2:**
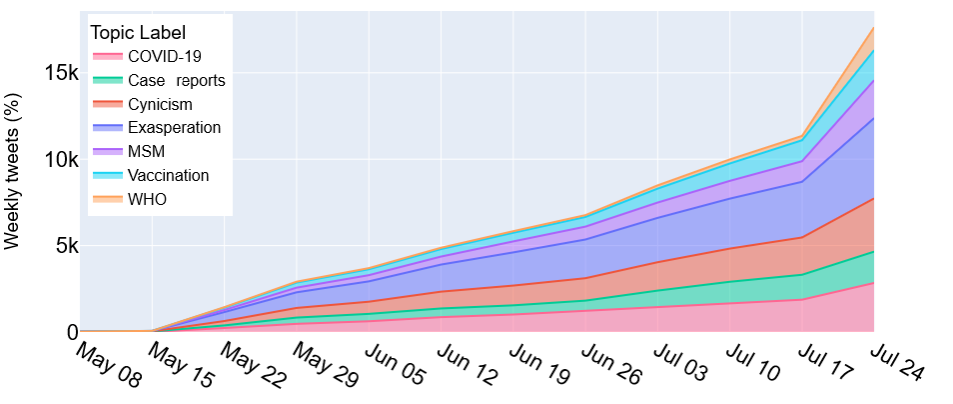
Cumulative message volume per cluster showing initial high volume of messages related to expressions of “Exasperation,” “Cynicism,” inaccurate comparison with “COVID-19,” and inaccurate information with respect to “vaccinations,” only later followed by official WHO announcements. The listed dates correspond to the final day of each week. MSM: men who have sex with men; WHO: World Health Organization.

We outlined the distribution of tweets per cluster over time in Figure 2. As mentioned, dominant discourse topics included expressions of exasperation with repeated health crises (COVID-19 followed by mpox) and politicization of the mpox outbreaks. After an initial period of public reaction, we observed the emergence of accurate health messaging from federal outlets, including the CDC, FDA, National Institutes of Health, and others. Note, however, that over time a sizable number of tweets originated from or consisted of references to official case reports and WHO communications, which may indicate the success and virality of public health messaging.

## Discussion

### Overview

This study characterized longitudinal Twitter dialogue pertaining to the 2022 global mpox outbreak. As the first major public health emergency following the COVID-19 pandemic, this work sought to confirm ongoing research about mpox through misinformation and information overload domains. Broadly, our results largely support the proverb, “a lie will travel halfway across the world before truth has managed to put her boots on,” specifically regarding the incongruent ratio between uninformed and misinformed mpox perspectives and accurate messaging from major health outlets. We contextualize our findings below.

### Misinformed Content Largely Predated Accurate Health Messaging From Official Outlets

As suggested in our findings, people may be more inclined to instinctively react to major news cycles before doing research on a topic. For example, our longitudinal analysis documented that, well before the WHO and other public health entities addressed the mpox outbreak, comments and posts about the earliest cases and the disease had already spread widely across the Twittersphere. This phenomenon of sharing and commenting in real time is largely referred to in the economic literature as “nowcasting” [42]. Nowcasting involves predicting the present or the near future based on indicators of consumer behavior or economic health from the past, and as such, is increasingly used to understand human behavior and decision-making [43]. Additionally, nowcasts can serve as early warnings to anticipate shock events driven by natural occurrences or experiments, such as the perceived severity of the mpox infection on public health. However, accurate nowcasting is contingent on the assumption that these predictions are based on reliable information. Unfortunately, our analysis indicates that the earliest discussions on mpox were largely guided by potentially uninformed views, highlighting the importance of fact-based information dissemination during public health emergencies.

Our data reveals that much of the information shared on the internet during the initial stages of the mpox outbreak was either disconnected from facts, politically biased, or contained humor and other irrelevant content. This phenomenon could be explained through the lens of the spiral of silence theory, which suggests that people influence one another’s willingness to express opinions through social interaction [44]. Alternatively, it may also be attributed to echo chambers and curated feeds, where individuals only interact with others who share similar views. Moreover, early content may reflect opinions rooted in controversies and public exhaustion related to the COVID-19 pandemic or unique features of the mpox disease, such as its initial association with MSM communities. As evident in the conspiracy, MSM, and exasperation topics, people were more likely to share lighthearted perspectives about mpox or downplay its potential severity. Nonetheless, this highlights important lessons about public perception of health topics and how to effectively communicate critical information amidst the abundance of web-based noise.

### Retweet Activity Supports the Need for Rapid Health Messaging

Our findings suggest that accurate health messaging can be effective in engaging the public. Specifically, our analysis showed that after the WHO declared an emergency, accurate topics related to mpox and mitigation strategies emerged and spread effectively on Twitter. The WHO cluster had the most retweets (average 73.54 retweets), followed by the MSM cluster (average 60.61 retweets). Within the MSM cluster, there were 2 types of tweets: those that incorrectly referred to mpox as a disease that exclusively affects marginalized portions of the US population (eg, tweet: “It only affects gay people, we have nothing to worry about”) and those that referred to specific mitigation strategies for the MSM community (eg, tweet: “If you identify as part of the LGBT community, a vaccine is available to combat Mpox”). Similarly, the case reports cluster, which was the third most retweeted cluster, also exhibited inaccurate content (eg, tweet: “Dude, these reports don’t matter. It’s not real”). However, we observed a greater number of people discussing the importance of these reports rather than dismissing them as irrelevant (eg, tweet: “Check out current MPox trends in [redacted], good to know and to stay informed!”).

Our findings appear to contradict some research that has demonstrated the challenges public health and social media entities face in monitoring and curbing the spread of misinformation related to COVID-19, such as the “Scamdemic” conspiracy theory and ongoing fake news about COVID-19 origins and vaccine mistruths [19]. Despite the consistent presence of noise across topics, we consistently observed pockets of accurate or helpful content related to mpox. This was especially evident after the WHO declared a global emergency and more case reports were disseminated on the internet. This observation may reflect the ongoing tension between misinformation and the release of accurate information, where the presence of accurate information may encourage people to share news more widely. Future research should consider experimental approaches to news sharing.

### Beyond Our Findings: Recommendations for Social Media Surveillance During Health Crises

In a systematic review of social media–driven misinformation, misinformed content was most prevalent regarding smoking and vaping, yet large proportions of misinformation were also observed in studies related to vaccines and other noncommunicable diseases [45]. In the same study, the authors likewise observed misinformation to be most prevalent on social networking website Twitter—the platform where data for our study originated; however, all commonly used social networking websites are also prone to misinformation risk. Recommendations to counter misinformation risk on social media are mixed [46-48]. However, previous research suggests that to address misinformation effectively, all angles of misinformation risk and spread must be considered, including message-related, source-related, receiver-related, and context-related factors [49]. As such, we offer 2 recommendations in contribution to this literature, including from digital surveillance and stigma-response perspectives.

First, from a computational and surveillance perspective, we recommend that public health institutions adopt a “situational awareness” approach to web-based messaging in which (1) social media is monitored in real-time using advances in natural language processing, computational psychology, and artificial intelligence to detect the dissemination of information that does not align with public health objectives, and (2) such information is proactively countered or augmented with messaging specifically designed to ensure the widest and most timely possible dissemination of crucial public health information [50,51]. In fact, social media platforms like Twitter offer a promising avenue to stem the distribution of false claims, such as real-time corrections, crowdsourced fact-checking, and algorithmic tagging [52]. However, we acknowledge such efforts rest on the ability of governmental public health agencies to adopt a proactive stance with respect to social media messaging and to maintain productive relations with computer scientists, public health scholars, and social media companies and leaders. Beyond surveillance, we also recommend further research into stigma-informed pandemic preparedness. Indeed, Logie [53] argues that a greater conceptual framework is needed, guided by lessons learned from HIV, COVID-19, and mpox. By examining stigma associated with the mpox outbreak, the HIV epidemic of the 1980s and 1990s, and pandemic information overload attributed to COVID-19, we stand to gain insight into potential messaging campaigns that transcend stigma and stereotypes. Greater digital surveillance would similarly ensure messages reach people at faster rates.

### Limitations

This study is subject to limitations we aim to address in future research. First, our analysis represents themes embedded within a fraction of the mpox Twitter data set. While it is possible to run more exploratory analyses with the entire corpus, using latent Dirichlet allocation topic models, for example, we strongly felt this more precise approach to computational thematic generation provided a more resounding portrait of discourse surrounding mpox over time. Second, we also acknowledge that we did not perform a full, in-depth qualitative review of these tweets. Though such an analysis may add nuance to our findings and discussion, our aim with this paper was to apply a novel methodological pipeline to quickly identify pockets of discourse that may be problematic or harmful amid another public health emergency. As such, we only applied a cursory qualitative review of these tweets to ensure all members of our team interpreted topics similarly. Future researchers should strongly consider replicating our findings using traditional qualitative inquiry, which can be used as a validation metric for our analytic pipeline.

### Conclusions

The global mpox outbreak offered another case study in public health response amid a global public health emergency. Although mpox infection rates never paralleled those during COVID-19, the anxieties experienced by people still reeling from pandemic-related trauma offered insight into how people’s social reactions and public health response have evolved since then. Our findings reveal a large presence of misinformed perspectives about mpox, including those at risk for infection, disbelief at the potential severity of mpox, and simple apathy toward another public health emergency.

## Abbreviations

BERT: Bidirectional Encoder Representations From Transformers
CDC: Centers for Disease Control and Prevention
FDA: Food and Drug Administration
MSM: men who have sex with men
PCA: principal component analysis
RQ: research question
S-BERT: Sentence Bidirectional Encoder From Transformers
UMAP: Uniform Manifold Approximation
WHO: World Health Organization

## Data Availability

All tweet IDs and code used to harvest tweets and produce the analysis and figures used in this study are available at [54].
